# *Acanthamoeba polyphaga*-Enhanced Growth of *Mycobacterium smegmatis*

**DOI:** 10.1371/journal.pone.0029833

**Published:** 2012-01-11

**Authors:** Otmane Lamrabet, Felix Mba Medie, Michel Drancourt

**Affiliations:** Unité de Recherche sur les Maladies Infectieuses et Tropicales Emergentes, UMR CNRS 6236 IRD 3R198, IFR48, Méditerranée Infection, Aix-Marseille Université, Marseille, France; Hopital Raymond Poincare - Universite Versailles St. Quentin, France

## Abstract

**Background:**

*Mycobacterium smegmatis* is a rapidly-growing mycobacterium causing rare opportunistic infections in human patients. It is present in soil and water environments where free-living amoeba also reside, but data regarding *M. smegmatis*-amoeba relationships have been contradictory from mycobacteria destruction to mycobacteria survival.

**Methodology/Principal Findings:**

Using optic and electron microscopy and culture-based microbial enumeration we investigated the ability of *M. smegmatis* mc^2^ 155, *M. smegmatis* ATCC 19420^T^ and *M. smegmatis* ATCC 27204 organisms to survive into *Acanthamoeba polyphaga* trophozoites and cysts. We observed that *M. smegmatis* mycobacteria penetrated and survived in *A. polyphaga* trophozoites over five-day co-culture resulting in amoeba lysis and the release of viable *M. smegmatis* mycobacteria without amoebal cyst formation. We further observed that amoeba-co-culture, and lysed amoeba and supernatant and pellet, significantly increased five-day growth of the three tested *M. smegmatis* strains, including a four-fold increase in intra-amoebal growth.

**Conclusions/Significance:**

Amoebal co-culture increases the growth of *M. smegmatis* resulting in amoeba killing by replicating *M. smegmatis* mycobacteria. This amoeba-*M. smegmatis* co-culture system illustrates an unusual paradigm in the mycobacteria-amoeba interactions as mycobacteria have been mainly regarded as amoeba-resistant organisms. Using these model organisms, this co-culture system could be used as a simple and rapid model to probe mycobacterial factors implicated in the intracellular growth of mycobacteria.

## Introduction

Mycobacteria are mycolic-acid containing, high GC% bacterial organisms belonging to the phylum *Actinobacteria*. They are recovered from soil and fresh water environments where free-living amoeba (FLA) are also living [1], [2], [3]. Co-isolation of mycobacteria and FLA collected from such environmental sources has been reported [4], [5]. Several experiments further demonstrated the ability of most environmental mycobacteria to survive in the amoebal trophozoites and to further reside into the amoebal cysts [6], [7], [8]. We recently showed that this holds true also for some of the *Mycobacterium tuberculosis* complex mycobacteria [9]. FLA have been therefore regarded as “Trojan horses” for such amoeba-resistant mycobacteria. Indeed, intra-amoebal survival has been demonstrated for 37 different *Mycobacterium* species and intra-amoebal surviving became a dogma for amoeba-mycobacteria interactions except for *Mycobacterium bovis* BCG which is killed by the FLA *Acanthamoeba castellanii* [8] and *Mycobacterium canettii* which bypasses amoebal encystement [9].

Amoeba-resistant mycobacteria include both slow-growing mycobacteria, i.e. mycobacteria sub-culturing over more than seven days and fast-growing mycobacteria which produce visible colonies in less than seven days [10]. Whereas fast-growing mycobacteria are comprised of both harmless organisms and opportunistic pathogens, slow-growing mycobacteria are comprised of some of the most successful bacterial human pathogens such as *M. tuberculosis* complex organisms causing tuberculosis [11], *Mycobacterium leprae* causing leprosy [12] and *Mycobacterium ulcerans* causing the Buruli ulcer [13]. Although several experimental studies have demonstrated the interactions of slow-growing mycobacteria, such as *Mycobacterium avium* complex members, with amoebae [6], [8], [9], [14], the interactions of fast-growing mycobacteria with amoebae remain poorly understood [14], [15], [16]. For example, conflicting results have been published regarding *Mycobacterium smegmatis*, ranging from its survival in the amoeba [15], [16] to its destruction by amoebae [14], [17].

*M. smegmatis* is the prototypical species of the so-called *M. smegmatis* group, which also contains *Mycobacterium wolinskyi* and *Mycobacterium goodii* [18]. Organisms of this group have seldom been associated with human infection, including orthopedic device infection and bacteremia [19], [20]. In the present work, we utilized *M. smegmatis* as a model organism to study the interactions of fast-growing mycobacteria with *Acanthamoeba polyphaga* which, together with *Acanthamoeba castellanii*, is one of two FLA routinely used to probe bacteria-FLA interactions [21] at large and more specifically mycobacteria-FLA interactions [22].

## Materials and Methods

### Mycobacterium strains

*M. smegmatis* mc^2^ 155 (ATCC 700084; a gift from Stéphane Canaan, Laboratoire d'Enzymologie Interfaciale et Physiologie de la Lipolyse CNRS UPR 9025, Marseille, France), *M. smegmatis* ATCC 19420^T^ and *M. smegmatis* ATCC 27204 purchased from German collection of microorganisms and cell cultures (DSMZ, Braunschweig, Germany) were used in this study. *M. smegmatis* organisms were cultured in Middelbrook 7H9 liquid medium (Sigma-Aldrich Logistic Gmbh, Lyon, France) and sub-cultured in Middlebrook and Cohn 7H10 agar (Becton Dickinson, Le Pont de Claix, France) at 37°C. Under these culture conditions, the three *M. smegmatis* strains yielded smooth colonies within three days.

### Microscopic detection of *A. polyphaga* infected with mycobacteria

*A. polyphaga* Linc-AP1 strain (a gift from T. J. Rowbotham, Public Health Laboratory, Leeds, United Kingdom) was grown at 28°C for 4 days in 150-cm^3^ culture flasks (Corning, New York, USA) containing 30 mL of peptone-yeast extract-glucose (PYG) broth. When average amoeba concentration reached 5×10^5^ cells/mL, amoebae were centrifuged at 500 g for 10 min and the pellet was suspended twice in 30 mL Page's modified Neff's Ameoba Saline (PAS) (Solution A-NaCl 1.20 g; MgSO_4_.7H_2_O 0.04 g; Na_2_HPO_4_ 1.42 g; KH_2_PO_4_ 1.36 g/100 mL of glass distilled water. Solution B-CaCl_2_.2H_2_O 0.04 g/100 mL of distilled water. Amoeba saline, 10 mL of solution A+10 mL of solution B+980 mL distilled water). Liquid medium-cultured *M. smegmatis* organisms were washed twice with PBS and the pellet was suspended in PAS. This inoculum was strongly vortexed to minimize mycobacterial clumping and the inoculum was determined by optic microscopy counting after Ziehl-Neelsen staining. Ten milliliters of the amoebal suspension in PAS (10^5^ amoeba/mL) were inoculated with 10^6^ mycobacteria/mL to achieve a MOI of 10 mycobacteria/amoeba. As controls, *A. polyphaga* and *M. smegmatis* were cultured separately in PAS. After incubation for 6 h at 32°C, the co-culture was washed three times with PAS to remove any remaining extracellular or adherent mycobacteria, and it was incubated in 10 mL PAS for 5 days at 32°C. After gentle shaking and cytocentrifugation at 100 g for 10 min, mycobacteria were detected inside amoebal trophozoites by Ziehl-Neelsen staining. Also, the presence of viable mycobacteria inside amoebal trophozoites was documented by sub-culturing. At 0, 24, 48, 72, 96 and 120 h time points, *A. polyphaga* monolayer were lysed with 0.1% Sodium dodecyl sulfate (SDS) (Sigma-Aldrich Logistic Gmbh) for 30 min and passed through a 26-gauge needle to ensure complete lysis of the amoebae. The lysate (100 µL) was plated onto 7H10 agar and incubated for four days at 37°C to determine the number of colonies (CFU) of intracellular *M. smegmatis*. The viability of amoeba, with and without bacteria, was done using Trypan Bleu coloration 0.4% (Sigma-Aldrich, Taufkirchen, Germany) and counting in the Glasstic slide chamber (HycoR, Garden Grove, California USA). Experiments were done in triplicate.

### Encystment of infected amoeba

Fifty milliliters of a 48-hour amoebal co-culture (concentration, 5×10^5^ amoebal cells/mL of PAS) were put in a 175-cm^3^ culture flask (Corning) and infected with 5 mL (concentration, 10^7^ mycobacteria cells/mL of PAS) of *M. smegmatis* suspension in PAS for 6 hours (time point, 0). The co-culture was washed twice with PAS to remove any remaining extracellular or adherent mycobacteria and it was incubated in 50 mL PAS for 5 days. In parallel, at different time points after infection (each 24 hours), ten milliliters of co-culture was taken, the supernatant was discarded and the amoebal monolayer was rinsed twice with encystment buffer (0.1 M KCl, 0.02 M Tris, 8 mM MgSO_4_, 0.4 mM CaCl_2_, 1 mM NaHCO_3_) before being incubated (at 32°C for 3 days) in fresh encystment buffer (0.1 M KCl, 0.02 M Tris, 8 mM MgSO_4_, 0.4 mM CaCl_2_, 1 mM NaHCO_3_). As control, *A. polyphaga* was cultured in encystment buffer. The process of excystment was verified by light microscopic examination of Ziehl-Neelsen smears. After 3 days, the number of cysts and trophozoites at different time points was determined by microscopic observation.

Moreover, the cysts corresponding to the time point 0 were then centrifuged at 1,000 g for 10 min and washed three times with PAS before using it for electron microscopic observation. Experiments were done in triplicate.

### Culture of *M. smegmatis* with amoeba debris

*A. polyphaga* and *M. smegmatis* were prepared as described before. After washing with PAS, 10 mL of *A. polyphaga* cells suspension (∼5×10^5^ amoeba/mL) were lysed (1 min at liquid nitrogen and 1 min at 37°C for three times) and centrifuged at 800 g for 10 min. 10^3^ mycobacteria/mL was separately incubated with amoeba lysis pellet and supernatant for 5 days at 32°C. *M. smegmatis* were observed in the culture at each time point by Ziehl-Neelsen staining. As controls, *M. smegmatis* were cultured in PAS. Experiments were done in triplicate.

### Ultrastructural study

Amoebal cysts and monolayers inoculated with mycobacteria were washed twice with sterile PAS to eliminate non-ingested mycobacteria. Samples were fixed in 2% glutaraldehyde and 0.1 M cacodylate buffer overnight, then in 2% glutaraldehyde and 0.33% acroleine in 0.07 M cacodylate buffer for one hour. After washing in 0.2 M cacodylate buffer, the preparation was post-fixed in 1% osmium tetraoxide in 0.1 M potassium ferrycyanure for one hour and dehydrated in an ascending series of ethanol concentrations, up to 100% ethanol. The samples were then successively incubated (for 45 min) in a 3∶1, 2∶2, 1∶3 (vol/vol) ethanol-Epon suspension, then in 100% Epon overnight with continuous shaking. Samples were embedded in an Epon 812 resin (Fluka, St Quentin Fallavier, France) and then incubated for three days at 60°C. Ultrathin sections (70 nm) were cut from the blocks using an ultracut microtome (Reichert-Leica, Marseille, France) before being deposited on Formvar-coated copper grids (Sigma-Aldrich). Ultrathin sections were stained for 10 min with 5% uranyl acetate and lead citrate before being examined using a transmission electron microscope (Morgani 268D; Philips, Eindhoven, Netherlands).

## Results

### *M. smegmatis* - A. polyphaga trophozoites co-culture

**Figure 1:**
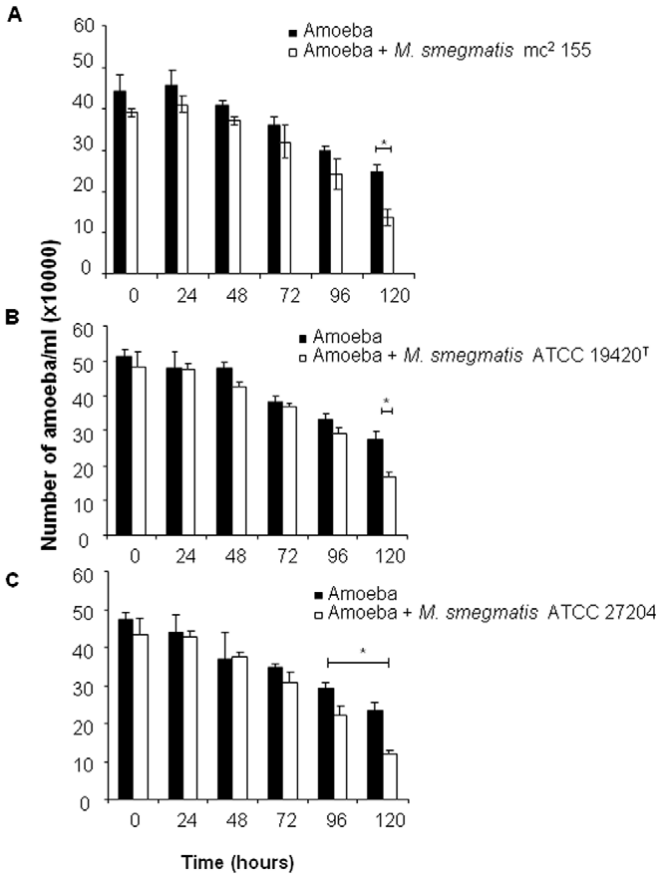
Amoeba increases the growth of *M. smegmatis*. Counting of amoeba alive with and without *M. smegmatis* mc^2^ 155 (A), *M. smegmatis* ATCC 19420^T^ (B) and *M. smegmatis* ATCC 27204 (C) in PAS. Asterix represent significant variation (p≤0.05). Each bar represents the mean of triplicate wells, and the standard errors are represented by error bars.

**Figure 2:**
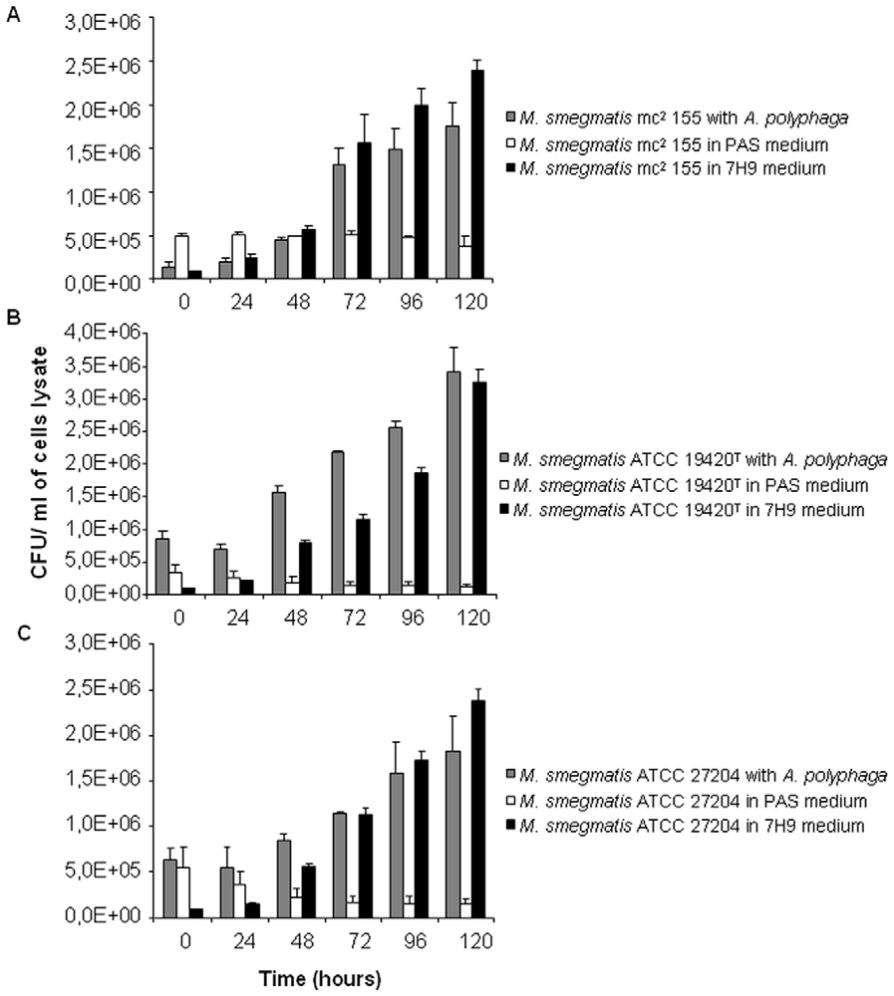
Growth of *M. smegmatis* within *A. polyphaga* trophozoites. *M. smegmatis* co-cultures with free-living amoeba *A. polyphaga* (gray bar) and alone in PAS medium (white bar) and in 7H9 complete medium (black bar). Three *M. smegmatis* organisms were tested: (A) *M. smegmatis* mc^2^ 155, (B) *M. smegmatis* ATCC 19420^T^ and (C) *M. smegmatis* ATCC 27204. Each bar represents the mean of triplicate wells, and the standard errors are represented by error bars.

**Figure 3:**
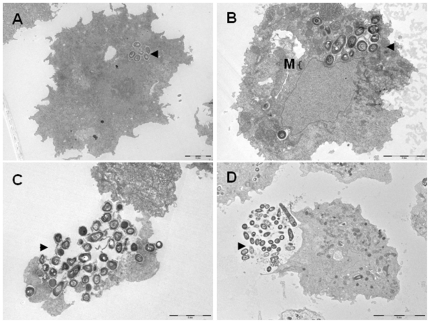
*M. smegmatis* is internalized into amoeba. Transmission electron-microscopy observation of *M. smegmatis* mc^2^ 155 (▸) co-cultivated with *A. polyphaga* trophozoites at (A) 0 hour, (B) 48 hours, (C) 72 hours and (D) 120 hours **m**: mitochondria. Scale bar: 2 µm (A, B, C) and 5 µm (D).

**Figure 4:**
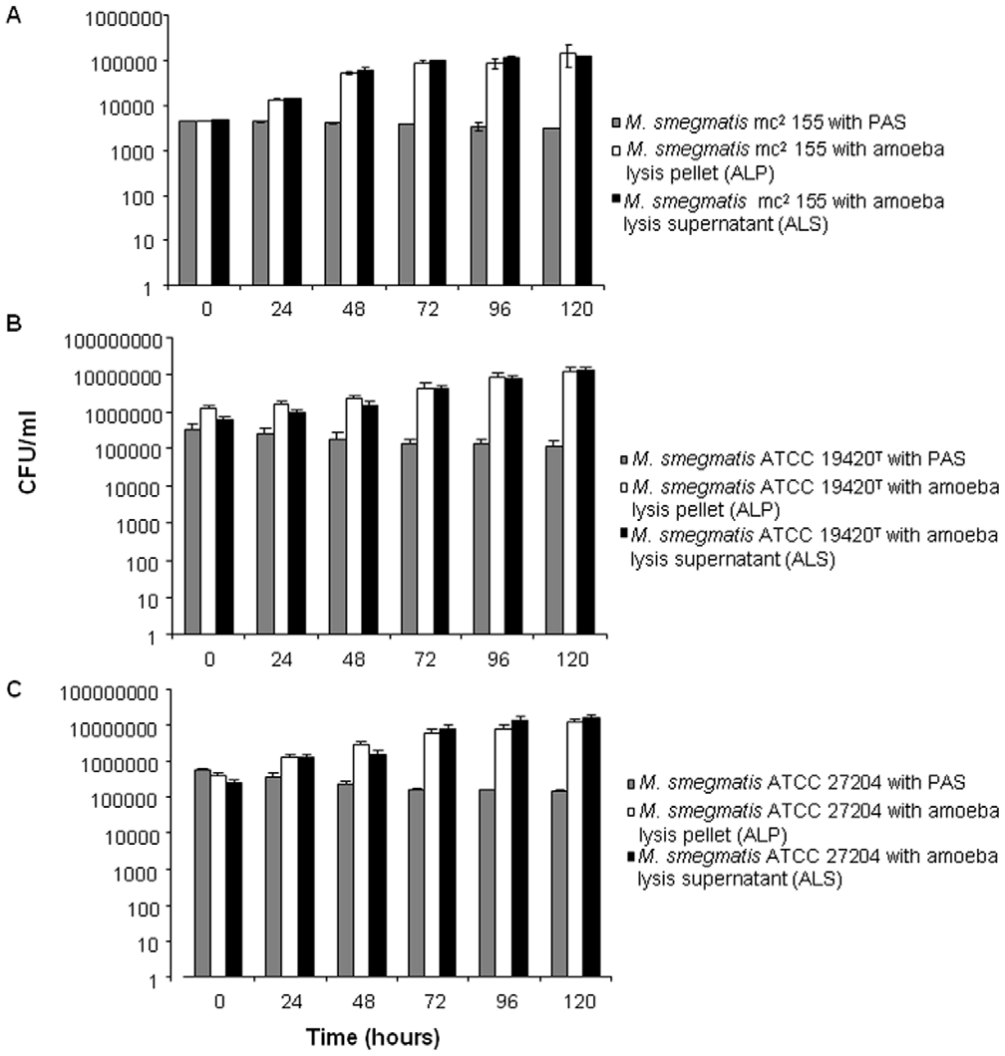
Growth of *M. smegmatis* in the presence of amoeba lysis. Three *M. smegmatis* organisms were tested: (A) *M. smegmatis* mc^2^ 155, (B) *M. smegmatis* ATCC 19420^T^ and (C) *M. smegmatis* ATCC 27204. *M. smegmatis* strains cultured with amoeba lysis pellet (white bar) and supernatant (black bar). PAS medium was used as negative control (gray bar). Each bar represents the mean of triplicate wells, and the standard errors are represented by error bars.

We first observed that the number of both non-infected and infected *A. polyphaga* trophozoites incubated into PAS at 32°C decreased over the time with the number of infected-amoeba decreasing significantly more than the number of non-infected amoeba (p≤0.05) at day four of co-culture for *M. smegmatis* ATCC 27204 and at day five of co-culture for *M. smegmatis* mc^2^ 155 and *M. smegmatis* ATCC 19420^T^, in triplicate experiment (Figure 1). In parallel, we observed that the three tested *M. smegmatis* strains survived but did not multiply over five-day incubation in PAS at 32°C (Figure 2). At six-hour incubation, *M. smegmatis* mc^2^ 155-*A. polyphaga* co-culture yielded 72% infected amoeba presenting at least one vacuole containing mycobacteria (Figure 3). Such vacuoles were surrounded by several mitochondria and displayed morphological features consistent with mycobacterial division, i.e. two organisms tightly attached by one extremity into a single vacuole (Figure 3). For the three tested *M. smegmatis* strains, quantification of colony forming units (CFU) co-cultured with amoeba indicated a significant increase (p<0.05) in the number of mycobacteria organisms starting at day 2 (Figure 2). To understand whether this significant increase in the growth of *M. smegmatis* co-cultured with *A. polyphaga* necessitated viable amoeba, we further cultured each one of the three tested *M. smegmatis* strains in PAS enriched in an amoeba lysis pellet (ALP) or an amoeba lysis supernatant (ALS). Regardless of the *M. smegmatis* strain, we observed that growth of *M. smegmatis* organisms was significantly increased (p≤0.05) by the addition of ALP or the addition of ALS to the PAS (Figure 4).

### Interaction of *M. smegmatis* mc^2^ 155 with *A. polyphaga* cysts

**Figure 5:**
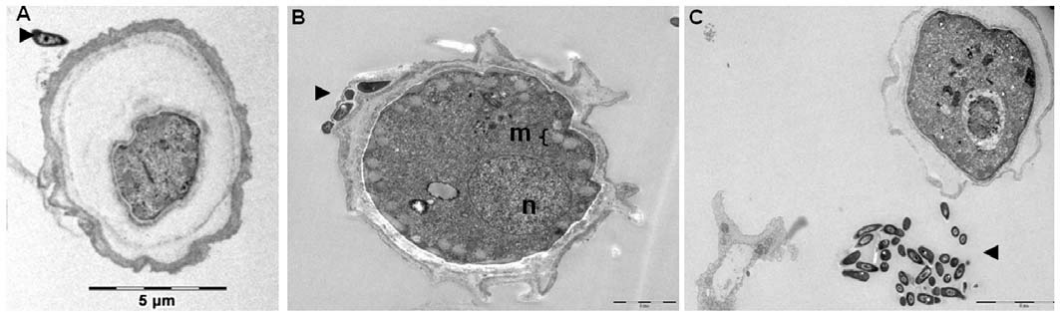
Transmission electron-microscopy observation of *A. polyphaga* cysts. (A) The mature form of cyst. *M. smegmatis* mc^2^ 155 (▸) exit from *A. polyphaga* pre-cyst (B) and present in the outside of pre-cyst (C); **n:** nucleus, **m:** mitochondria. Scale bar: 5 µm (A, C) and 2 µm (B).

We further infected *A. polyphaga* trophozoites with *M. smegmatis* mc^2^ 155 organisms for 6 hours, and then incubated in encystement buffer for 3 days noted as days 0–3. A sample was then taken every 24 hours and microscopic examination disclosed cystic formation in 43% of *M. smegmatis*-infected amoebae at day 0 (6 hours of infection); 38% at day 1; 19% at day 2 and 8% at day 3. Non-infected, negative control amoeba yielded 46% encystment at day 0; 52% at day 1; 71% at day 2 and 78% at day 3. This difference in the percentage of encysted amoeba was statistically significant from day 0 to day 3 in triplicate experiment (p≤0.05). Electron microscopy further identified mature cysts by the presence of condensation of indistinct components implicated in the metabolism and replication in the middle of this form (Figure 5A), and pre-cysts identified by the presence of the nucleus and mitochondria scattered into the cytoplasm (Figure 5B). Careful electron microscopy observation of 500 cysts formed at day 3 failed to reveal any *M. smegmatis* organisms into *A. polyphaga* cysts (Figure 5C). In one case only the *M. smegmatis* organism was observed to have moved from the endocyst of a pre-cyst present in the earlier phase of encystation after three-day encystment (Figure 5B). Experimental encystment of *A. polyphaga* co-culture yielded no intracystic mycobacteria after a three-day encystement.

## Discussion

The data presented in this study were interpreted as authentic because negative controls remained negative in each experimental step. In this work, two model organisms have been used in order to set-up a standardized co-culture system. Moreover, similar results were obtained when testing three different strains of *M. smegmatis*, including one type strain as well as *M. smegmatis* mc^2^ 155 (ATCC 700084). Indeed, *M. smegmatis* mc^2^ 155 strain, the only *M. smegmatis* strain with available genome sequence, has particular parietal features which may not be found in other *M. smegmatis* strains. This could have biased results. We herein show that this was not the case. Moreover, *M. smegmatis* mc^2^ 155 has known genetics and it has been previously used in 37/46 (80%) studies dealing with *M. smegmatis* – macrophage/amoeba interactions (Table S1). In addition, *M. smegmatis* mc^2^ 155 is commonly used as a model strain for the cloning genes from harmful mycobacteria [17]. Likewise, *A. polyphaga* has been extensively used for studying amoeba-mycobacteria interactions [6]. The co-culture system herein reported is therefore a standardized system which could be reproduced in other laboratories. We observed that *M. smegmatis* organisms readily penetrated into *A. polyphaga* trophozoites, a reproducible result obtained by using a low (1∶10) multiplicity of infection (MOI). We further observed that such intra-amoebal mycobacteria survived into *A. polyphaga* trophozoites, a fact documented by microscopic observations. Previously published data regarding the *M. smegmatis*-amoeba relationships have been conflicting: some studies reported that *M. smegmatis* survived within *A. castellanii* [15], [16], whereas other studies found the opposite [14], [17]. These discrepancies may be explained by the fact that a 30-minute amoeba-*M. smegmatis* co-culture used in some studies may be insufficient for the mycobacteria to penetrate into the amoeba. Thus, our data expand the previous demonstration of intra-amoebal surviving of *M. smegmatis* in amoeba *A. castellanii* to another species of amoeba, *A. polyphaga*.

We further observed that *M. smegmatis* organisms multiply within amoeba during the time of the experiment and that *M. smegmatis* lysed the amoeba at the 4–5 days p.i. peak of its intra-amoebal growth. Amoebal lysis has been previously reported for the rapidly growing *Mycobacterium chelonae*, *Mycobacterium abscessus*, *Mycobacterium monacense* and *Mycobacterium neoaurum* [23]. Also, 63 of 454 non-mycobacterial strains isolated from water yielded complete and rapid lysis of amoebae [23]. These bacteria were organisms closely related to *Clostridium haemolyticum*, *Methylobacterium sp.*, *Pseudomonas aeruginosa* and *Bradyrhizobium japonicum* [24], [25].

Interestingly, we further observed that pelleted debris of lysed amoeba and the supernatant of such lysed amoeba also significantly enhanced the growth *M. smegmatis* mycobacteria, regardless of the strain under study. This observation reminds recent observations made when co-culturing *Salmonella enterica* Typhi with *A. castellanii* [21] and suggests that amoeba contain one or several currently uncharacterized growth-promoting factors or nutriments for *M. smegmatis*. Determining such factors was beyond the scope of present study, but further culture-based experiments incorporating fractions of amoeba supernatant are warranted to precise the nature of these factors.

We further observed that *M. smegmatis* moved out of the *A. polyphaga* pre-cyst before its maturation; this observation extended previous data found for other rapidly growing mycobacteria such as *Mycobacterium septicum* [6]. This observation contrasts with previous observations that slowly growing mycobacteria survived within the amoebal exocyst [26]. It was observed that 92% of *M. avium*-infected trophozoites evolved into mature cysts whereas we observed that only 8% of *M. smegmatis*-infected trophozoites produced mature cysts at the same time [26]. Accordingly, forced encystment of *M. smegmatis*-infected *A. polyphaga* amoeba yielded no mycobacteria in the cysts. Taken together, these data suggest that fast-growing mycobacteria rapidly escape the encystment to infect new amoebal trophozoites. Interestingly, we recently observed that *M. canettii* was the only tested *M. tuberculosis* complex member to by-pass the *A. polyphaga* encystement [9]. Exactly as for *M. smegmatis*, *M. canettii* also massively invaded the amoeba host [9].

**Figure 6:**
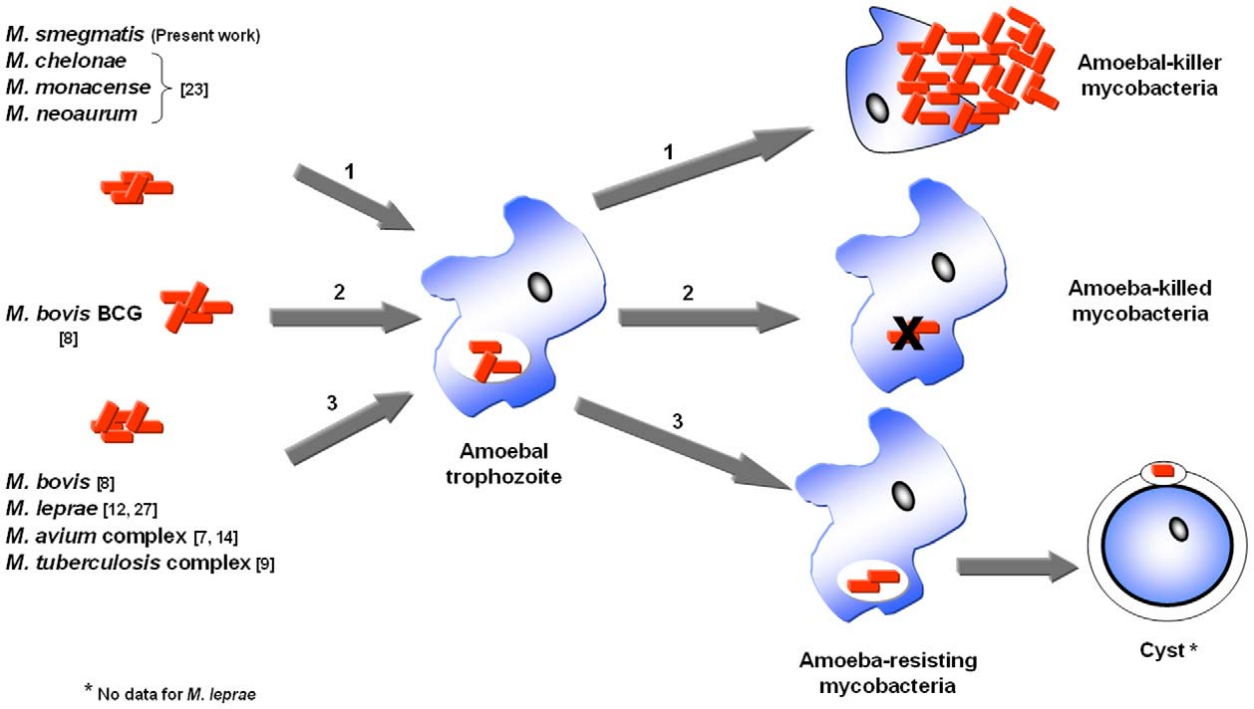
Different forms of amoeba-mycobacteria interactions.

Previously published findings [15], [16] coordinated with herein presented results, suggest that rapidly growing mycobacteria should be regarded as amoeba-killing mycobacteria contrary to slowly growing mycobacteria (Figure 6). Indeed, most previous experimental studies of amoebae-mycobacteria interactions focused on slowly growing mycobacteria (Table 1). It has been observed that these species, such as *M. bovis* [8], *M. tuberculosis* [9], *M. leprae* [12], [27], *Mycobacterium xenopi* [26] and members of the *M. avium* complex [7], [14], can survive and/or multiply within trophozoites.

**Table 1:** Described interactions of rapid and slow-growing mycobacteria with FLA.

Bacterial species	Growing mycobacteria		Described interaction with protozoa	References
	Rapid	Slow		
*Mycobacterium abscessus*	✓		IC survival and multiplication (Ap)	[23]
*Mycobacterium avium*		✓	IC multiplication (Ac), IK survival (Ap)	[14]
*Mycobacterium avium subsp. avium*		✓	IC multiplication (Ap), IK survival (Ap)	[6], [7]
*Mycobacterium avium subsp. paratuberculosis*		✓	IC multiplication (Ap), IK survival (Ap)	[31]
*Mycobacterium aurum*	✓		IC multiplication (Ap), IK survival (Ap)	[6]
*Mycobacterium bohemicum*		✓	IC and IK survival (Ap)	[6]
*Mycobacterium bovis*		✓	IC survival (Ac)	[8]
*Mycobacterium bovis BCG*		✓	No survival (Ac)	[8]
*Mycobacterium chelonae*	✓		IC survival and multiplication (Ap)	[23]
*Mycobacterium fortuitum subsp. fortuitum*	✓		IC multiplication (Ac)	[15]
*Mycobacterium fortuitum*	✓		IC multiplication (Ac), IC and IK survival (Ap)	[6]
*Mycobacterium gastri*		✓	IC and IK survival (Ap)	[6]
*Mycobacterium goodii*	✓		IC and IK survival (Ap)	[6]
*Mycobacterium gordonae*		✓	IC and IK survival (Ap)	[6]
*Mycobacterium gilvum*	✓		?	-
*Mycobacterium immunogenum*	✓		IC and IK survival (Ap)	[6]
*Mycobacterium intracellulare*		✓	IC and IK survival (Ap)	[6]
*Mycobacterium kansasii*		✓	IC multiplication (Ac), IC and IK survival (Ap)	[6], [32]
*Mycobacterium lentiflavum*	✓	✓	IC and IK survival (Ap)	[6]
*Mycobacterium leprae*		✓	IC survival (*A. culbertsoni*)	[12], [16]
*Mycobacterium mageritense*	✓		IC and IK survival (Ap)	[6]
*Mycobacterium malmoense*		✓	IC and IK survival (Ap)	[6]
*Mycobacterium marinum*		✓	IC multiplication (Ac), IC and IK survival (Ap)	[6], [33]
*Mycobacterium massiliense*	✓		IC and IK survival (Ap)	[18]
*Mycobacterium mucogenicum*	✓		IC and IK survival (Ap)	[6]
*Mycobacterium peregrinum*	✓		IC and IK survival (Ap)	[6]
*Mycobacterium phlei*	✓		IC and IK survival (Ac)	[15]
*Mycobacterium porcinum*	✓		IC and IK survival (Ap)	[6]
*Mycobacterium septicum*	✓		IC and IK survival (Ap)	[6]
*Mycobacterium scrofulaceum*		✓	IC multiplication (Tp), IK survival (Tp)	[34]
*Mycobacterium simiae*		✓	IC and IK survival (Ap), IC survival (Ac)	[6], [15]
*Mycobacterium smegmatis*	✓		IC survival and multiplication (Ap)	Present work
*Mycobacterium szulgai*		✓	IC and IK survival (Ap)	[6]
*Mycobacterium tuberculosis*		✓	IC survival (Ap)	[9]
*Mycobacterium terrae*		✓	IC and IK survival (Ap)	[6]
*Mycobacterium tusciae*		✓	IC and IK survival (Ap)	[6]
*Mycobacterium ulcerans*		✓	IC survival (Ac, Ap)	[15], [35]
*Mycobacterium xenopi*		✓	IC multiplication (Ap), IK survival (Ap)	[9], [26]

IC, intracellular; IK, intracyst; Ap, *Acanthamoeba polyphaga*; Ac, *Acanthamoeba castellanii*; Tp, *Tetrahymena pyriformis*.

We previously proposed that amoeba are a training field for macrophage resistance of mycobacteria [28]. Several studies used amoeba to investigate the phagocytosis and intracellular survival mechanisms of pathogens including *Legionella pneumophila* [29], *Yersinia pseudotuberculosis* [23] and *P. aeruginosa* [30]. *M. smegmatis* has been used to develop genetic engineering of mycobacteria and the *M. smegmatis*-amoeba co-culture developed here could therefore be used as a simple and rapid first-line system to scan mycobacterial factors implicated in the intracellular growth of mycobacteria.

In conclusion, the spectrum of interactions between amoeba and environmental mycobacteria may be wider than previously appreciated. It includes mycobacteria such as *M. leprae* surviving in amoeba [12], [27], mycobacteria such as *M. avium* and *M. tuberculosis* multiplying in amoeba as opportunistic organisms [9], [7], [14] and mycobacteria such as *M. chelonae* [23] and *M. smegmatis* killing the amoeba (Figure 6).

## Supporting Information

Table S1 **The** ***M. smegmatis*** **strain used in 46 published studies on** ***M. smegmatis*** **– macrophage/amoeba interactions.**
(XLS)
